# Peri‐Implant Diseases: Enhanced Antibacterial Photodynamic Therapy

**DOI:** 10.1002/cre2.70146

**Published:** 2025-05-19

**Authors:** Hanna Lähteenmäki, Tommi Pätilä, Ismo T. Räisänen, Rauni Kalliala, Timo Sorsa

**Affiliations:** ^1^ Department of Oral and Maxillofacial Diseases University of Helsinki and Helsinki University Hospital Helsinki Finland; ^2^ Department of Pediatric Heart Surgery and Organ Transplantation, New Children's Hospital Helsinki University Helsinki Finland; ^3^ Division of Periodontology, Department of Dental Medicine Karolinska Institutet Huddinge Sweden

**Keywords:** aMMP‐8, antibacterial photodynamic therapy, oral hygiene, peri‐implant disease

## Abstract

**Objectives:**

The number of dental implants is constantly on the rise, and inflammation of their surrounding tissues is an increasing problem. In this randomized controlled trial, we investigated the effects of antibacterial dual‐light photodynamic therapy (aPDT) in peri‐implant disease in reduction of inflammation in the peri‐implant tissues.

**Material and Methods:**

Forty patients with newly diagnosed peri‐implant disease were recruited. The diagnoses were established clinically and by radiological bone loss (RBL). Dual‐light aPDT was provided by indocyanine green mouth rinsing and 50:50, 405 nm, and 810 nm light applicator. The treatment group (*n* = 20) was randomized to use aPDT treatment at home for 4 weeks. The treatment was carried out once daily for 2 weeks, then twice daily for the next 2 weeks. The control group (*n* = 20) continued enhanced self‐care during the study period. Traditional measurement methods around the implant were measured at the beginning, at 2 weeks and at 4 weeks.

**Results:**

During the study period, VPI decreased in both groups. In the treatment group BOP decreased from mean (SD) 4.7 ± 1.3 to 1.8 ± 1.6, *p* < 0.0001, but no change was observed in the control group (3.5 ± 2.3 to 3.0 ± 2.3, *p* = 0.39). In the aPDT treatment group aMMP‐8 decreased from mean (SD) 100 ± 41 to 72 ± 38, *p* = 0.027, but not in the control group (86 ± 54 to 86 ± 60, *p* = 0.38).

**Conclusions:**

Regularly applied dual‐light aPDT reduces inflammation in the dental implant adjacent tissues. Dual‐light aPDT approach holds promise as an effective home care tool for implant patients.

## 1 Introduction

Peri‐implant disease in its early exert form can bemanaged reversible manner. With meticulous biofilm control, the peri‐implant tissues have a chance to heal (Heitz‐Mayfield et al. 2020). Dual‐light antibacterial photodynamic therapy (aPDT) is a novel and modern technique, which provides remarkable antibacterial effectiveness against dysbiotic biofilms (Lähteenmäki et al. 2022; Nikinmaa et al. 2020; Nikinmaa, Podonyi, et al. 2021). This method is available for home‐use, and regular application has demonstrated markedly improved treatment results when used as an adjunct to periodontitis treatment (Pakarinen et al. 2022; Petelin et al. 2015; Konopka and Goslinski 2007; Nikinmaa et al. 2020; Sculean et al. 2020). In this study we investigated the effect of regular dual‐light aPDT home‐use in peri‐implant disease, when combined to scaling and root planning.

## 2 Materials and Methods

### 2.1 Study Design and Participants

Forty patients (January 2023) were enrolled in this prospective controlled trial and were calculated to be adequate to detect the effect size. The study protocol was approved by the ethical committee of the Hospital District of Helsinki and Uusimaa (HUS/16329/2022) in accordance with the Helsinki Declaration. The inclusion criteria were (1) implant patients arriving for a maintenance visit at a dental clinic (Hammasklinikka Kruunu, Tampere, Finland); (2) a diagnosis of peri‐implant healthy, peri‐implant mucositis or peri‐implantitis by a dentist according to the definitions described below; and (3) a written informed consent. The exclusion criteria included (1) smoking; (2) age under 25; (3) antibiotic treatment within 6 months; (4) the presence of a major physical limitation or restriction that prohibits the hygiene procedures used in the study protocol. A single infected implant with screw crown in each patient was selected for the study. All participants gave informed consent before participating in the study. Randomization was done using a sealed envelope method. The patients were divided into two groups to receive either hygiene instructions (control group) or hygiene instructions with adjunct dual‐light aPDT treatment (treatment group). In conjunction with their routine oral care practices at home, the treatment group participants were instructed to incorporate the dual‐light aPDT treatment in their home routine. They were required to use this method once daily for the initial 2‐week period, and then twice daily for the following 2 weeks. The total length of the treatment period was 4 weeks. We hypothesized that antibacterial photodynamic therapy improves tissue health by reducing inflammation without side effects. Monitored by measuring dental plaque, gingival bleeding, implant probing depths and aMMP‐8 levels (Lähteenmäki et al. 2022) (Prevention of dental implant diseases ClinicalTrials.gov ID NCT05871229). This study has complied with the CONSORT guidelines protocol.

### 2.2 Dental Examination

The dental examinations were performed at the beginning of the study, at 2 weeks and at 4 weeks. An oral clinical investigation at each time point included an assessment for six sites of the implant of interest with a millimeter‐grade Ball‐Tip Screening probe, WHO, AEEP23/WHOBX (American Eagle Manufacturing Co., New Bern, NC, USA). Bleeding on probing (BOP) was measured at 6 points around the implant of interest. The deepest implant probing pocket depth (PPD) was measured in millimeters from the gingival margin to the base of the implant pocket and visible plaque index (VPI) (scaling 0–3 on all surfaces). A digital intra‐oral radiograph was acquired using Sirona Heliodent plus (Dentsply Sirona, New York, NY, USA), and Soredex Digora Optime (KaVo Dental GmbH, Biberach, Germany) software was used for the analysis of alveolar bone loss to define peri‐implantitis. Clinical photographs were shot at all time points with an iPhone 8 mobile phone 12 MP camera with f/1.8 aperture, six‐element lens, optical stabilization, and Wide Color capture deployment (Apple Inc., Cupertino, CA, USA). The diagnostic definition of peri‐implant health and disease was based on the criteria described by Consensus report of workgroup 4 of the 2017 World Workshop on the Classification of Periodontal and Peri‐Implant Diseases and Conditions (Berglundh et al. 2018; Renvert et al. 2018).

### 2.3 Peri‐Implant Sulcus Fluid (PISF) Collection and Analysis Samples

The quantification of the biomarker aMMP‐8 (in nL) in peri‐implant sulcular fluid (PISF) was carried out using the ImplantSafe test and the ORALyzer digital aMMP‐8 reader device (Dentognostics, Jena, Germany). To collect PISF samples, a collection strip was inserted into the peri‐implant pocket at the sulcus point for a duration of 30 s. Following the manufacturer's instructions, the strip was then placed in an elution liquid for a 5‐min period. After this incubation time, the elution tube was gently swirled a few times. For the ImplantSafe test, a dipstick was immersed into the elution liquid for 15 s and subsequently inserted into the ORALyzer device for quantitative reading (Lähteenmäki et al. 2022; Sorsa et al. 2017).

### 2.4 Oral Hygiene Protocol by Photodynamic Treatment

**Figure 1:**
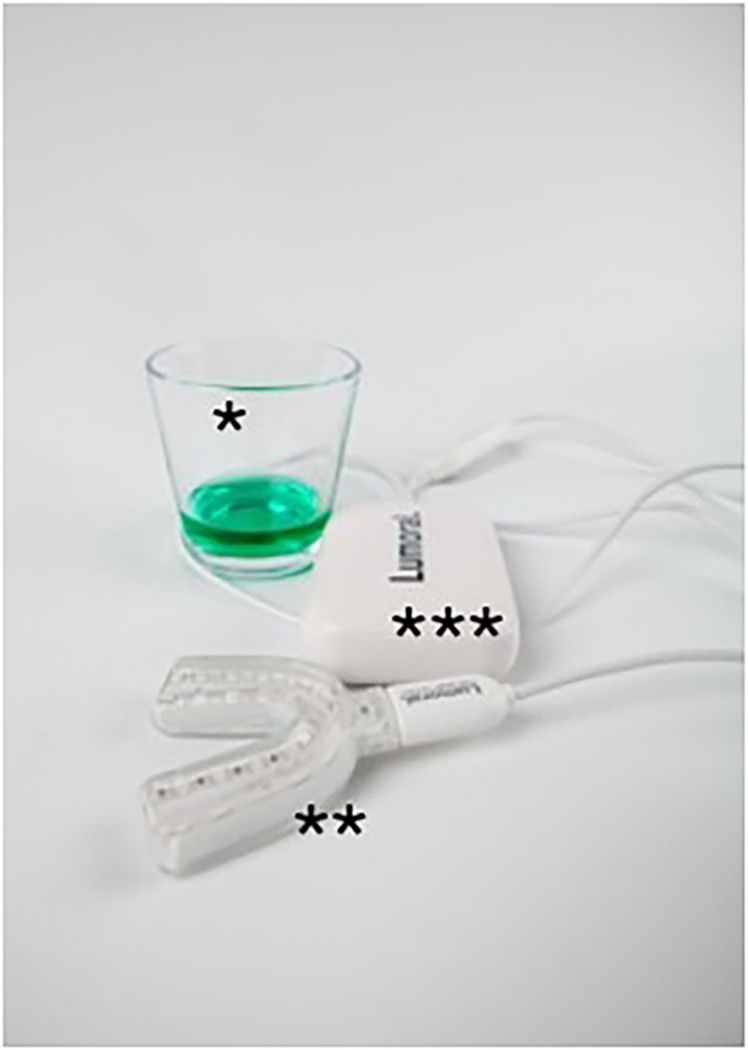
The dual‐light aPDT device.

Dual‐light antibacterial photodynamic therapy (aPDT) is an advanced, noninvasive method for enhancing oral biofilm control by combining two complementary light‐based mechanisms. The treatment begins with a mouth rinse containing indocyanine green (ICG), a photosensitizer that selectively binds to dental plaque. This is followed by light activation using a mouthguard‐like device that simultaneously emits 405 nm antibacterial blue light (aBL) and 810 nm near‐infrared (NIR) light. The blue light exerts a direct antimicrobial effect by exciting endogenous chromophores within bacteria, while the NIR light activates the ICG to produce reactive oxygen species that further disrupt the plaque biofilm. The dual‐light approach targets both surface bacteria and deeper biofilm layers, providing enhanced antibacterial efficacy without the use of antibiotics, and is well suited for regular home use (Figure 1).

Indocyanine green photosensitizer was provided effervescent tablet containing 7 mg of indocyanine green (ICG), dissolved in 30 mL of water to create a mouthwash with an ICG concentration of 250 μg/mL (Lumorinse; Koite Health LTD, Espoo, Finland). This mouthwash is swirled in the mouth for 1 min, allowing the ICG to adhere to dental plaque before light exposure. Light activation was performed with a LED light applicator assembled in the form of a mouthguard. The light applicator simultaneously emits aBL and NIR in 50:50 ratio. The device uniformly illuminates the entire set of teeth, as the 48 LEDs illuminate both upper and lower dental arches (Lumoral; Koite Health LTD, Espoo, Finland). After the mouth rinse, the light applicator is inserted into the mouth, and during a 10‐min session, it delivers a radiant exposure of 40 J/cm² of light. When used in conjunction with the ICG mouthwash, this product provides a dual action of aBL and aPDT targeted at dental plaque.

### 2.5 Statistical Methods

GraphPad version 9 (GraphPad Software, La Jolla, CA, USA) was used for statistical analyses and graph generation. Wilcoxon signed‐rank tests were applied for nonparametric comparisons of paired groups, and paired *t*‐tests were used to analyze differences in continuous variables between measurement points. Categorical data were analyzed using contingency tables and Fisher's exact test. Statistical significance was defined as a two‐tailed *p*‐value < 0.05. Power analysis was performed using previously collected data.

Sample size was calculated using the ClinCalc Sample Size Calculator based on the design of a previous study, with an alpha error of 5% and a Type II error of 20% (corresponding to 80% statistical power). This yielded a required sample size of 18 patients per group. To account for potential dropouts, a total of 20 patients per group were recruited for this pilot study.

## 3 Results

All 40 study participants completed the whole protocol. There were no significant differences in the prevalence of comorbidities between the groups, see the patient demographics in Table 1. The year of placement of the implants ranged from 1997 to 2021, with a median of 6.0 years since placement. In the treatment group the subject ages ranged from 46 to 74 years, with 11 implants in the mandibulae and 9 in the maxilla. Eighteen implants were produced by Nobel Biocare and there was no information about two implants. In the control group subject ages ranged from 35 to 82 years, with 15 implants being from the mandibulae and 5 being from the maxilla. Eighteen implants were produced by Nobel Biocare® and there was no information about two implants. The year of placement of the implants ranged from 1997 to 2021, with a median of 6.0 years since placement. The patient characteristics are presented in Table 1.

**Table 1:** The patient characteristics.

Condition/variable	Control croup (*n* = 20)	Treatment group (*n* = 20)	*p* value
Gender, *n, %*			
Female	11 (55%)	15 (75%)	*p* = 1.000
Male	9 (45%)	5 (25%)	
Age, mean ± SD	66.75 ± 11.29	65.65 ± 8.2	*p* = 1.000
Diabetes	2 (10%)	3 (15%)	*p* = 1.000
Heart disease	5 (25%)	4 (20%)	*p* = 1.000
Asthma	1 (5%)	1 (5%)	*p* = 1.000
Tooth			
Upper teeth	15 (75%)	11 (55%)	*p* = 0.627
Lover teeth	5 (25%)	9 (45%)	
Diagnose			
Peri‐implant mucositis	20 (100%)	16 (80%)	*p* = 0.742
Peri‐implantitis	0 (0%)	4 (20%)	

**Figure 2:**
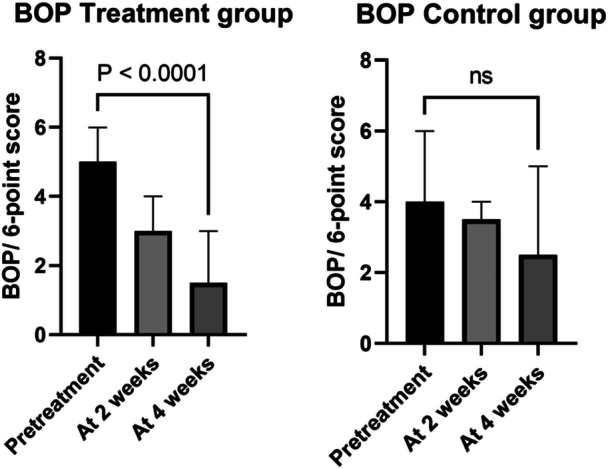
During the 4‐week study period, in the treatment group BOP decreased from mean (SD) 4.7 ± 1.3 to 1.8 ± 1.6, *p* < 0.0001, but no change was observed in the control group (3.5 ± 2.3 to 3.0 ± 2.3, *p* = 0.39).

**Figure 3:**
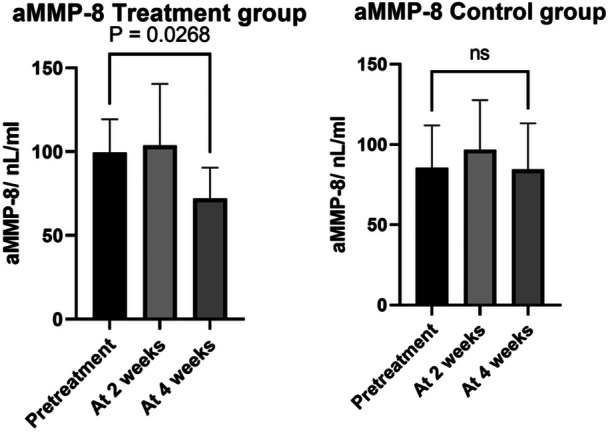
In the treatment group aMMP‐8 decreased from mean (SD) 100 ± 41 to 72 ± 38, *p* = 0.027, but not in the control group (86 ± 54 to 86 ± 60, *p* = 0.38).

**Figure 4:**
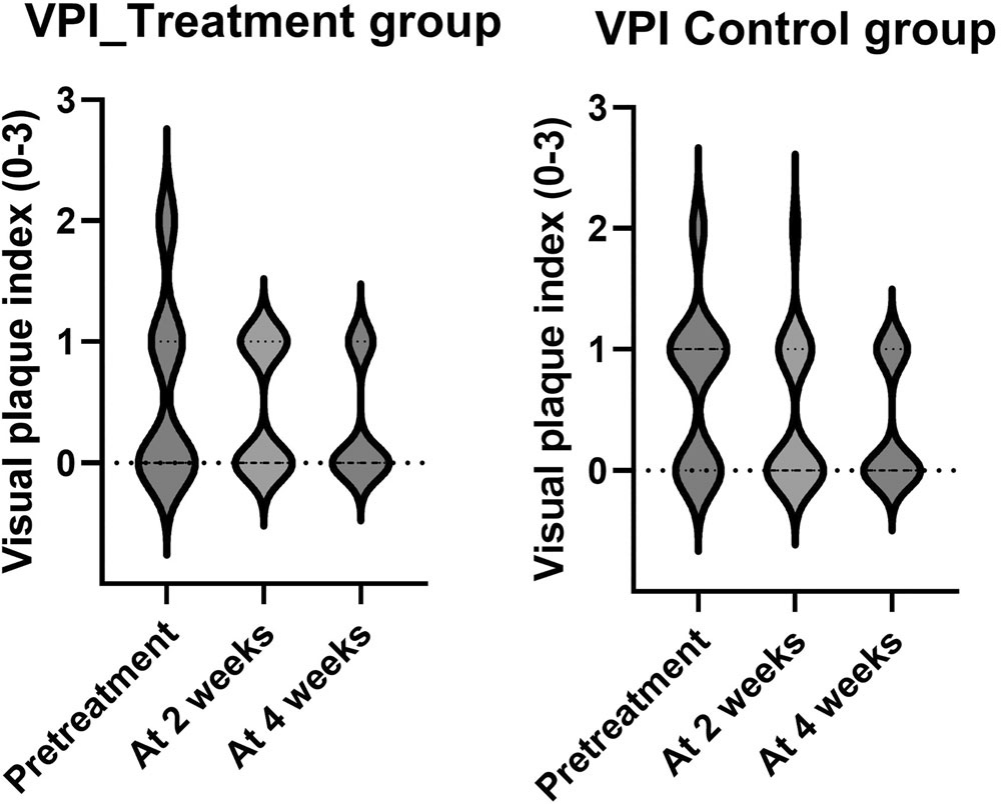
The Box‐violin charts describe the plaque reduction in the implants of interest. There was a statistically significant reduction of plague in both groups, and no difference between the groups.

During the 4‐week study period, in the treatment group BOP decreased from mean (SD) 4.7 ± 1.3 to 1.8 ± 1.6, *p* < 0.0001, but no change was observed in the control group (3.5 ± 2.3 to 3.0 ± 2.3, *p* = 0.39), see Figure 2. In the treatment group aMMP‐8 decreased from mean (SD) 100 ± 41 to 72 ± 38, *p* = 0.027, but not however in the control group (86 ± 54 to 86 ± 60, *p* = 0.38), see Figure 3. There was a statistically significant reduction of plaque in both groups, with no statistical difference between the groups, see Figure 4. Reduction in the probing depth of the deepest peri‐implant pocket was observed in four cases in the treatment group, and zero cases in the control group (*p* = ns.), see Table 2.

**Table 2:** The deepest pocket measured around the implant of interest in the A. treatment group and in the B. control group.

A. Treatment Group			B. Control Group		
At pretreatment	At 2 weeks	At 4 weeks	At pretreatment	At 2 weeks	At 4 weeks
8	8	8	4	5	5
3	2	2	5	5	5
4	4	4	5	5	4
4	4	4	3	3	3
3	3	3	2	2	2
4	4	4	4	4	4
4	5	5	4	4	4
3	3	3	2	2	2
3	3	3	5	5	5
6	5.5	5.5	4	4	4
3	3	2	3	3	3
7	7	7	2	2	2
3	3	3	5	5	5
6	6	6	2	2	2
3	3	3	4	4	4
4	4	4	4	4	4
6	6	6	5	5	5
3	3	3	4	4	4
4	4	4	3	3	3
4	4	3	3,5	4	4

## 4 Discussion

This prospective controlled trial indicates that regularly applied dual‐light aPDT at home can be beneficial for patients with peri‐implant disease. We observed better oral hygiene in all participants, as shown by a decrease in visible plaque. However, the inflammatory measurements were markedly lower in the treatment group, including reduction in BOP and also decrease in aMMP‐8 measured from the PISF. There were some reduced pockets measured, but as expected, no significant differences were found between the groups. It's important to note that none of the participants received any professional clinical treatment during the study period. All these improvements were achieved through enhanced oral hygiene practices at home.

In this study, the health status of implant teeth was studied and monitored using traditional measurement methods as well as molecular testing aMMP‐8 methods. Enhanced inflammation of the tissues of implant teeth is quite common, so we wished to study of the effects of aPDT therapy on implant tissue health. The size of the data set can affect the outcomes of the study. Compared to many other fields, dental studies are typically small and complex (Faber and Fonseca 2014). In our previous study, the results already showed significant results in seven patients (Lähteenmäki 2022), and then a sample of twenty yielded similar results. These results provide robust, generalizable evidence that the aMMP‐8 enzyme test can be used for real‐time diagnosis of adjacent tissues to implant teeth and aPDT therapy has aspects affecting the health of implant tissues.

The aMMP‐8‐PoC test can be carried out to alert and detect active collagenolysis affecting the peri‐tissues of the implant (Sorsa et al. 2017). According to previous studies, aMMP‐8 PoC testing can be considered a reliable method for diagnosing the health status of implant tissue (Lähteenmäki et al. 2022). The study population in the control group had slightly more healthy implant tissue than the Lumoral group. This can cause a slight bias between the results of the study, since it is known that it is easier to treat healthy or peri‐mucositis patients. The improvement score in the Lumoral group was proportionally higher than in the control group.

This study hypothesis was that can dual light therapy reduce signs of inflammation in the mouth. We suggest a benefit of home‐applied dual‐light aPDT as the personalized medicine use in patients with peri‐implant disease. This new gamechanger anti‐inflammatory therapy can reduce the signs of inflammation, infection, and proteolysis in the oral cavity. A 30–60 J/cm^2^ dose of aPDT is effective in inactivating anaerobic bacteria. To our knowledge, no side effects exist for aPDT (Nikinmaa et al. 2020; Hentilä et al. 2021; Nikinmaa, Moilanen, et al. 2021; Lähteenmäki et al. 2022).

Dual‐light antibacterial photodynamic therapy operates through a combined treatment approach, which has demonstrated effectiveness in eradicating *Streptococcus mutans* (Nikinmaa et al. 2020). The method employs 405 nm aBL and 810 nm near‐infrared (NIR) light. While the NIR light is used with indocyanine green for antibacterial effects, it's also absorbed by the mitochondrial enzyme cytochrome‐c‐oxidase in eukaryotic cells. This absorption initiates downstream effects, including enhanced ATP production. This action, known as photobiomodulation, holds significant potential in addressing oral mucositis and has gained substantial recognition in clinical applications (National Institute for Health and Care Excellence (NICE) (2018)).

aPDT is safe to use without side‐effects. The supposed disadvantages can be the warming of the mouth due to the operation of the device; increased secretion of saliva and possible short‐term staining of the soft tissue surfaces of the mouth. The application of light and dyes to destroy microbial species in vitro has been reported for many years (Matsubara et al. 2008).

While laser‐applied aPDT demonstrates potency in antibacterial action, within their limitations, systematic reviews have reported only minor benefits in managing peri‐implant disease (Herrera et al. 2023). The disappointment in its efficacy may be attributed to the fact that many studies have administered the treatment infrequently, often limited to one or two applications (de Oliveira et al. 2007; Joseph et al. 2017). Notably, instances where aPDT has shown greater effectiveness have been in studies that implemented repetitive treatment sessions (Giannelli et al. 2015, 2018; Lähteenmäki et al. 2022). This underscores the substantial influence of treatment frequency on the observed response.

More studies and tools are needed for the success of peri‐implant treatments. The applicability of the aMMP‐8 chairside test in diagnosing the health status of the attachment tissues of implant teeth and in monitoring treatment offers a useful and more accurate diagnosing methods. New gamechanger oral medicine technologies brings opportunities to treat implant teeth and oral cavity at home even more efficiently than traditional dental care routines. However, further studies are warranted to define the effectiveness of treatment.

## Author Contributions

Hanna Lähteenmäki, Tommi Pätilä, and Timo Sorsa contributed to the study's conception and design. Hanna Lähteenmäki, Ismo Räisänen, and Tommi Pätilä contributed to the data acquisition, analysis, and interpretation. Hanna Lähteenmäki and Tommi Pätilä wrote the manuscript, and Rauni Kalliala and Timo Sorsa revised the manuscript. All authors reviewed and approved the final version of the manuscript.

## Ethics Statement

The study was conducted in accordance with the ethical principles of the Declaration of Helsinki. The study was approved by the ethics committee of the Hospital District of Helsinki and Uusimaa (HUS/16329/2022), and all participants provided written informed consent before enrollment.

## Conflicts of Interest

Tommi Pätilä is the Chairman of the board and owns stocks in Koite Health LTD. The company has filed patents and owns trademarks related to antibacterial dual light. Koite Health LTD develops, sells, and markets dual‐light antibacterial products for the prevention and treatment of dental and periodontal infections. Timo Sorsa is the inventor of the following patents: 1274,416‐patent US 5,652,223, 5,736,341, 5,864,632, 6,143,476, and US 2017/0023571A1 (issued June 6, 2019); WO 2018/060553 A1 (issued May 31, 201); 10,488,415 B2, Japanese Patent 2016‐554676; and South Korean patent 10‐2016‐ 7025378.

## Data Availability

The data sets from this study are available from the corresponding author upon reasonable request.
